# Unacylated ghrelin attenuates acute liver injury and hyperlipidemia via its anti-inflammatory and anti-oxidative activities 

**DOI:** 10.22038/IJBMS.2023.70831.15388

**Published:** 2024

**Authors:** Yating Gong, Beibei Qiu, Haotian Zheng, Xiangbo Li, Yifan Wang, Mengran Wu, Meixing Yan, Yanling Gong

**Affiliations:** 1 Department of Pharmacy, College of Chemical Engineering, Qingdao University of Science and Technology, Qingdao, China; 2 Department of Pathology, Feicheng Hospital Affiliated to Shandong First Medical University, Qingdao, China; 3 Department of Pharmacy, Qingdao Women and Children’s Hospital, Qingdao, China

**Keywords:** Anti-inflammatory, Anti-oxidative, Hyperlipidemia, Intraperitoneal injection, Liver injury, Unacylated ghrelin

## Abstract

**Objective(s):**

Liver injury and hyperlipidemia are major issues that have drawn more and more attention in recent years. The present study aimed to investigate the effects of unacylated ghrelin (UAG) on acute liver injury and hyperlipidemia in mice.

**Materials and Methods:**

UAG was injected intraperitoneally once a day for three days. Three hours after the last administration, acute liver injury was induced by intraperitoneal injection of carbon tetrachloride (CCl_4_), and acute hyperlipidemia was induced by intraperitoneal injection of poloxamer 407, respectively. Twenty-four hours later, samples were collected for serum biochemistry analysis, histopathological examination, and Western blotting.

**Results:**

In acute liver injury mice, UAG significantly decreased liver index, serum alanine aminotransferase (ALT), aspartate aminotransferase (AST), interleukin-6 (IL-6), and tumor necrosis factor-α (TNF-α), reduced malondialdehyde (MDA) concentration and increased superoxide dismutase(SOD) in liver tissue. NF-kappa B (NF-κB) protein expression in the liver was down-regulated. In acute hyperlipidemia mice, UAG significantly decreased serum total cholesterol (TC), triglyceride (TG), ALT, and AST, as well as hepatic TG levels. Meanwhile, hepatic MDA decreased and SOD increased significantly. Moreover, UAG improved the pathological damage in the liver induced by CCl_4_ and poloxamer 407, respectively.

**Conclusion:**

Intraperitoneal injection of UAG exhibited hepatoprotective and lipid-lowering effects on acute liver injury and hyperlipidemia, which is attributed to its anti-inflammatory and anti-oxidant activities.

## Introduction

As one of the most important organs, the liver plays a vital role in regulating physiological processes and participates in the metabolism, excretion, and storage of fatty acids and other macromolecules (1, 2). This organ is also responsible for scavenging metabolites and exogenous compounds and is vulnerable to toxic damage from these compounds and hyperlipidemia. Given the important functions of the liver, damage caused by exogenous compounds might result in serious consequences, characterized by varying degrees of liver damage (1, 2), ranging from steatosis to steatohepatitis, fibrosis, and necrosis (3, 4). Moreover, triglycerides and other fats may circulate and deposit in the liver, leading to fatty infiltration which ultimately increases the risk of cirrhosis and liver failure (5, 6). Therefore, liver disease is a global health problem, among which acute liver injury is closely related to acute hyperlipidemia and inflammation (7, 8). The prevention and treatment of liver injury and hyperlipidemia is an important step in the clinical treatment of liver disease (9). Therefore, it has become a research hotspot to develop an efficient and safe hepatoprotective and lipid-lowering drug to prevent or treat liver function injury and hyperlipidemia caused by various factors.

Ghrelin is a peptide containing 28 amino acids and the only natural ligand of the growth hormone secretin receptor (GHSR) (10). There are two main circulating forms for ghrelin: acylated ghrelin (AG) and unacylated ghrelin (UAG). Although UAG accounts for 80–90% of circulating ghrelin (11), initial reports termed UAG as an inactive peptide. This is mainly due to the inability of UAG to activate GHSR at the physiologic level (12) and the lack of an identified receptor or mediator that accounts for the activities of UAG. Nonetheless, the bioactivity of UAG should not be neglected. Several authors suggested that UAG could be metabolically active by counteracting the effects of AG on insulin secretion and glucose metabolism in healthy humans (13-15). Studies have shown that ghrelin may affect adipocyte metabolism. Ghrelin, as well as UAG, may act directly as antilipolytic factors on the adipose tissue through binding to a specific receptor which is distinct from growth hormone secretagogue type 1a (GHS-R1a) (16). The mitogenic and antiapoptotic actions of ghrelin in 3T3-L1 adipocytes have been demonstrated (17, 18). Moreover, UAG analogs can prevent oxidative stress-induced apoptosis by stimulating silencing the information regulator 2 related enzyme 1 (sirtuin1, SIRT1) signaling pathway (19, 20), thus mitigating the body injury. Exogenous administration of UAG reduced the circulatory ratio of AG/UAG, thereby attenuating HFD-induced hepatic steatosis. The possible mechanism of UAG might be involved in suppressing lipogenesis, stimulating fatty acid oxidation, preventing oxidative stress, inflammation, endoplasmic reticulum stress, and apoptosis (21). However, the possible intervention of UAG in acute liver injury and hyperlipidemia has yet not been revealed. The present study aims to observe the effect of exogenous UAG on acute liver injury and hyperlipidemia in mice. The possible mechanism based on anti-inflammatory and anti-oxidative activities is also revealed. This study is expected to provide a reference for expanding the application and development of UAG.

## Materials and Methods

***Animals***

A total of 96 male Kunming mice (20 g±2 g) were purchased from Qingdao Daren Fucheng Animal Husbandry Co, LTD. All mice were fed at 22±2 °C and 55±10% humidity with a 12:12-hr light-dark cycle. Experiments were conducted after one week of acclimation and approved by the animal ethics committee of Qingdao University of Science and Technology (approval number: ACQUST-2022-079), in accordance with the Guide for the Care and Use of Laboratory Animals. 

***CCl***_4_ ***-induced acute liver injury***

Forty-eight mice were randomly distributed into six groups (n=8): control group, liver injury group, UAG low dose group (50 µg/kg), UAG medium dose group (100 µg/kg), UAG high dose group (200 µg/kg), and bifendate group. Mice in the UAG group were intraperitoneally injected with UAG (NJPetide, Nanjing, China) at 8:00 a.m. every day for three consecutive days. Mice in the bifendate group were intraperitoneally injected with bifendate (200 mg/kg, Wanbang, Zhejiang, China). Mice in the other two groups were intraperitoneally injected with the same volume of saline. Three hours after the last administration, all mice in each group were intraperitoneally injected with 0.1% CCl_4_ (Xin Yu Biotech, Shanghai, China; dissolved in corn oil) except the control group with the same volume of corn oil. The volume of intraperitoneal injection was 0.1 mL/10 g body weight. 

***Poloxamer 407-induced acute hyperlipidemia***

Forty-eight mice were randomly divided into six groups (n=8): control group, hyperlipidemia group, UAG low dose group (50 µg/kg), UAG medium dose group (100µg/kg), UAG high dose group (200 µg/kg), and simvastatin group. Mice in the UAG group were intraperitoneally injected with UAG (NJPetide, Nanjing, China) at 8:00 a.m. every day for three consecutive days. Mice in the simvastatin group were intraperitoneally injected with simvastatin (6.7 mg/kg, Qingdao Jisskang Biotechnology, Shandong, China). Mice in the other two groups were intraperitoneally injected with the same volume of saline. Three hours after the last administration, all mice in each group were intraperitoneally injected with poloxamer 407 (300 mg/kg, Fengli jingqiu, Beijing, China) except the control group. The volume of intraperitoneal injection was 0.1 ml/10 g body weight. 

***Sample collection***

Twenty-four hours after the model establishment, the mice were weighed and samples were collected. Blood was collected from the eyeballs to separate serum which was kept in the -20 °C refrigerator. The abdominal cavity was cut to observe the appearance of the liver. Then the liver was removed, drained with filter paper, and weighed to calculate the liver index using the following formula:

 liver index=

A small piece of liver was taken and prepared into 10% homogenate using ice saline, a small piece of liver tissue was immersed in 10% paraformaldehyde to prepare a paraffin section, and another piece was stored at -80 °C.

***Biochemical analysis***

Serum levels of total cholesterol (TC), triglycerides (TG), alanine aminotransferase (ALT), aspartate aminotransferase (AST), hepatic TG, malondialdehyde (MDA), and total superoxide dismutase (SOD) concentration were determined by commercial colorimetric kits (Nanjing Jiancheng Bioengineering Institute, Nanjing, China). Serum interleukin-6 (IL-6) and tumor necrosis factor-α (TNF-α) were analyzed using ELISA kits (Solarbio, Beijing, China). All these measurements were in accordance with the manufacturers’ instructions.

***HE staining***

Histological morphology of the liver tissue was assessed via HE staining using paraffin-embedded slices. The photographs were taken under a microscope (Biological microscope type XSP-2CA, Shanghai, China) with the same magnification (×100).

***Western blot analysis***

The hepatic tissues were lysed using RIPA (Solarbio, Beijing, China) containing protease inhibitors, and the protein concentration was determined using bicycin chondrogenic acid (BCA, Solarbio, Beijing, China) protocol. Protein samples were denatured and then added to the gel sample wells which were subsequently transferred to a PVDF membrane (Millipore Corporation, USA). The primary antibodies to LPL (1:2000, Abcam, Shanghai, China), NF-κB (1:1000, Bioss, Beijing, China), β-actin (1:10000, Bioss, Beijing, China), and HRP conjugated goat anti-rabbit lgG (1:50000, Bioss, Beijing, China) were incubated. The chemiluminescence detection was performed using an ECL reagent (Solarbio, Beijing, China) and bands were developed with a gel imager (TANON-4600, Tianneng Technology, Shanghai, China). Specific bands were detected, analyzed, and quantified using the Image J Software package (v 1.44, Bethesda, Rockville, MD, USA). 

***Statistical analyses***

Data were processed using the SPSS17.0 software package with completely randomized designed one-way ANOVA followed by *post h*oc multiple comparisons using LSD or Dunnett’s T3 test. All values were expressed as the mean±standard deviation, and *P*<0.05 indicated a statistical significance.

## Results

***UAG alleviated CCl***_4_ ***-induced acute liver injury in mice***

**Figure 1:**
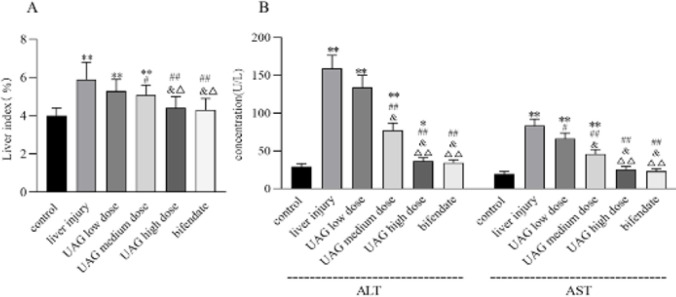
Effect of UAG on liver index (A) and aminotransferases (B) in mice with acute liver injury

As expected, intraperitoneal injection with CCl_4_ in mice induced an acute liver injury, manifested by elevated liver index as well as serum ALT and AST concentrations (liver injury group vs control group, *P*<0.01, Figure 1A and 1B). After intraperitoneally injected with UAG, the liver index, and concentrations of serum ALT and AST decreased significantly (vs Liver injury group, *P*<0.05 or 0.01, Figure 1A and 1B), showing a dose-dependent manner. High dose of UAG restored hepatomegaly and transaminases to normal levels, which was comparable to the effect of the positive control drug bifendate (Figures 1A and 1B). The results demonstrated a hepatoprotective activity of UAG on CCl_4_-induced acute liver injury in mice.

**Figure 2:**
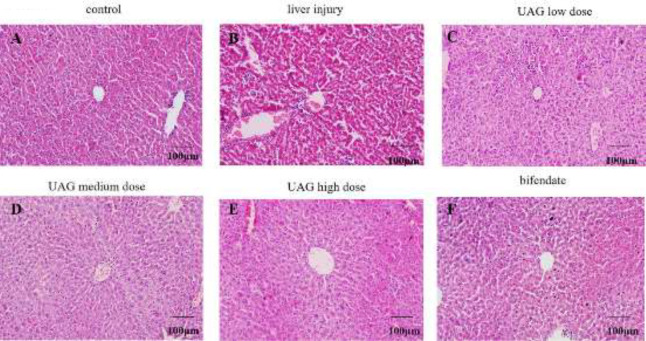
Effect of UAG on pathological liver injury in mice with acute liver injury. Bars:100 µm

Liver HE staining demonstrated the pathological changes in the liver. In the control group, the structure of the liver lobule was intact with orderly arranged hepatic cords (Figure 2A). Compared with the control group, the hepatic lobule structure in the liver injury group was blurred with disordered arranged hepatic cords. Cellular degeneration, necrosis, and inflammatory infiltration were observed locally (Figure 2B). After treatment with UAG, the above pathological changes were improved significantly with the increase of UAG dose (Figures 2C, 2D, and 2E). In the UAG high-dose group, the histopathological appearances in the liver showed no significant difference from the control group (Figure 2E). As expected, bifendate significantly alleviated acute liver injury induced by CCl_4_ (Figure 2F). 

**Figure 3:**
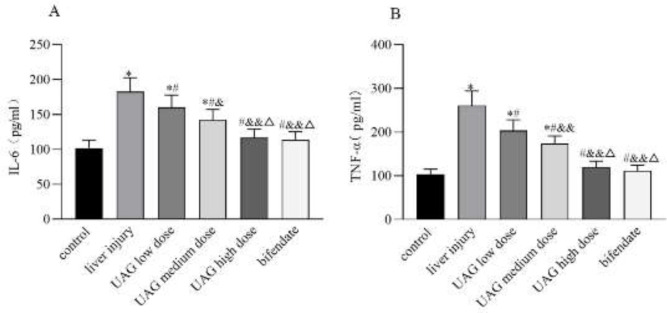
Effect of UAG on serum IL-6 (A) and TNF-α (B) in mice with acute liver injury

Compared with the control group, serum IL-6 and TNF-α of the mice in the liver injury group significantly increased, indicating that CCl_4_ induced inflammatory response therefore resulted in acute liver injury (*P*<0.01, Figures 3A and 3B). However, UAG intraperitoneally injected into mice significantly decreased serum IL-6 and TNF-α (vs the liver injury group, *P*<0.01, Figures 3A and 3B). The inhibition of the inflammatory response of UAG was beneficial to its hepatoprotective activity on CCl_4_-induced acute liver injury in mice. The anti-inflammatory effect of UAG increased significantly as the dose increased, among which the effect of high-dose UAG was similar to that of bifendate. 

**Figure 4:**
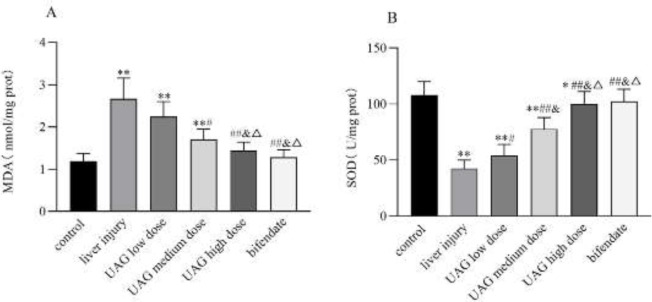
Effect of UAG on MDA (A) and SOD (B) in the liver of mice with acute liver injury

Compared with the control group, MDA level in the liver significantly increased (*P*<0.01, Figure 4A), while SOD concentration in the liver significantly decreased in the liver injury group (*P*<0.01, Figure 4B). The results suggested evidence of oxidative stress in the liver induced by CCl_4_. Meanwhile, the MDA level decreased (*P*<0.05 or 0.01, Figure 4A) while SOD concentration increased significantly (*P*<0.05 or 0.01, Figure 4B) in the UAG-treated group with different doses. Neither the UAG high dose group nor the bifendate group showed any significant difference from the control group (Figures 4A and 4B). These results suggested that the hepatoprotective effect of UAG might be related to the improvement of anti-oxidant capacity in acute liver injury mice induced by CCl_4_.

**Figure 5:**
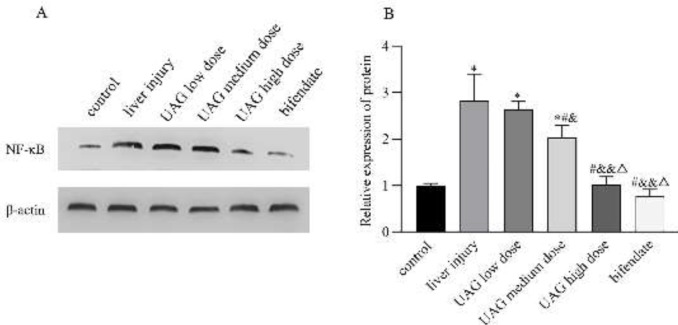
Effect of UAG on NF-κB expression (representative bands in A and quantitative analysis in B) in the liver of mice with acute liver injury

The results of western blot analysis showed that the expression of hepatic NF-κB in the liver injury group significantly increased compared with the control group (*P*<0.01, Figures 5A and 5B), suggesting that there might be activation of the hepatic inflammatory response signaling pathway leading to liver injury. Compared with the liver injury group, the expression of NF-κB significantly decreased after administration of different concentrations of UAG (*P*<0.01, Figures 5A and 5B). The expression level of NF-κB in the high-dose UAG group as well as in the bifendate group showed no significant difference from that in the control group (Figures 5A and 5B). The results indicated that UAG may down-regulate NF-κB expression, thereby reducing the production of inflammatory factors to achieve the hepatoprotective effect.

***UAG alleviated poloxamer 407-induced acute hyperlipemia in mice***

**Figure 6:**
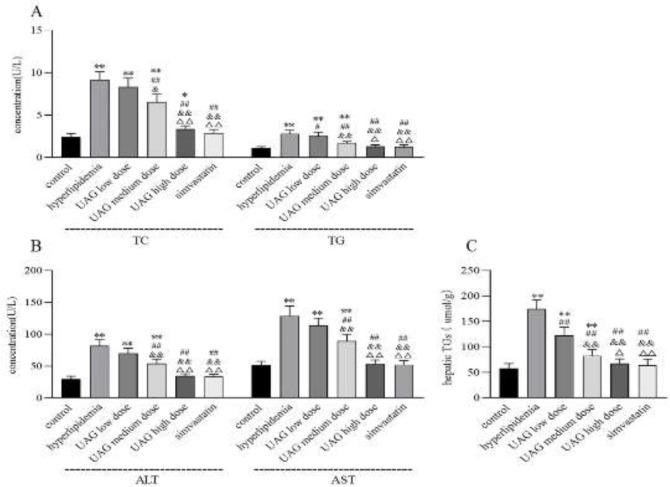
Effect of UAG on serum lipids (A), aminotransferases (B), and hepatic TG (C) in mice with acute hyperlipidemia

Intraperitoneal injection with poloxamer 407 in mice induced acute hyperlipidemia, characterized by elevated serum TC and TG levels (hyperlipidemia group vs control group, *P*<0.01, Figure 6A). Furthermore, there was also an elevation of ALT, AST, and hepatic TG in the hyperlipidemia group (vs control group, *P*<0.01, Figures 6B and 6C), indicating an injury in hepatic function and lipid metabolism. After intraperitoneal injection with UAG, the serum TC and TG levels, as well as ALT, AST concentrations, and hepatic TG decreased significantly (vs hyperlipidemia group, *P*<0.05 or 0.01, Figures 6A, 6B, and 6C), exhibiting a dose-dependent relationship. High dose of UAG restored the above parameters to normal levels (Figures 6A, 6B, and 6C), similar to the positive control drug simvastatin. The results demonstrated lipid-lowering and hepatoprotective activities of UAG on poloxamer 407-induced acute hyperlipidemia in mice.

**Figure 7:**
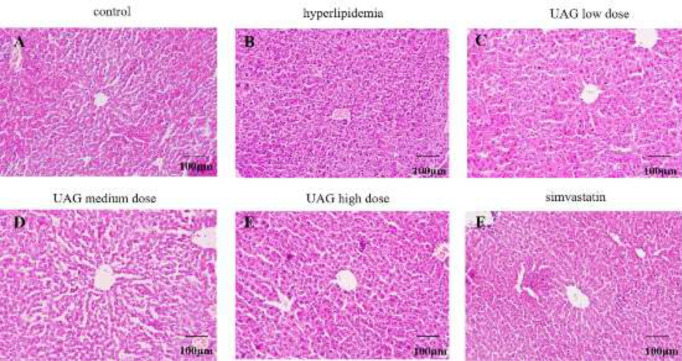
Effect of UAG on pathological liver injury in mice with acute hyperlipidemia

Liver HE staining showed that there were many fat vacuoles in the liver cells in the hyperlipidemia group, accompanied by disordered live lobes when compared with the control group (Figures 7B and 7A), indicating that poloxamer 407 resulted in hepatocyte steatosis. After treatment with UAG, the above pathological changes were improved significantly with the increase of the UAG dose (Figures 7C, 7D, and 7E). As expected, simvastatin significantly inhibited the hepatocyte steatosis induced by poloxamer 407 (Figure 7F). 

**Figure 8:**
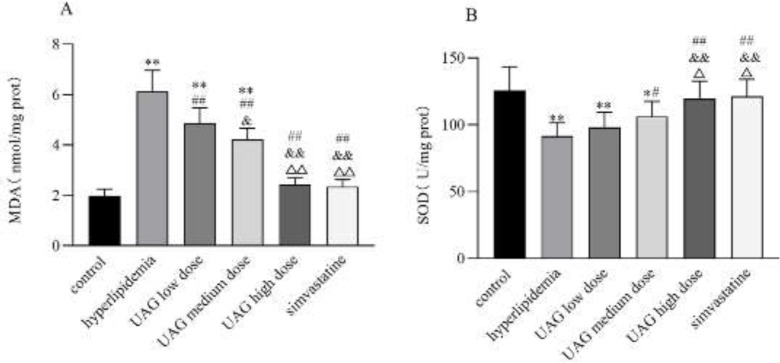
Effect of UAG on MDA and SOD in the liver of mice with acute hyperlipidemia

In the hyperlipidemia group, MDA level in the liver significantly increased (vs control group, *P*<0.01, Figure 8A), while SOD concentration in the liver significantly decreased (vs control group, *P*<0.01, Figure 8B). The results suggested evidence of oxidative stress in the liver induced by poloxamer 407. Meanwhile, MDA level decreased (vs hyperlipidemia group, *P*<0.01, Figure 8A) while SOD concentration increased significantly (vs hyperlipidemia group, *P*<0.05 or 0.01, Figure 8B) in the UAG treated group with different doses. The effect of high-dose UAG was similar to that of simvastatin, showing no significant difference with the control group (Figure 8A and 8B). These results suggested that the improvement in the anti-oxidant capacity of UAG was beneficial to the hepatoprotective effect in acute hyperlipidemia mice induced by poloxamer 407.

**Figure 9:**
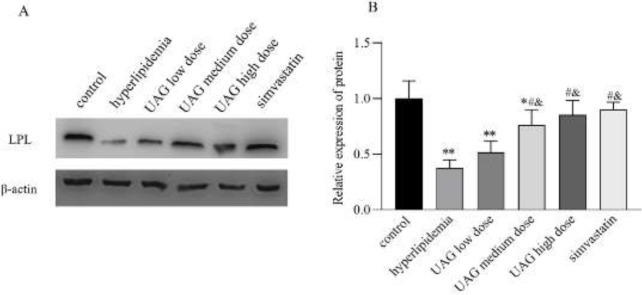
Effects of UAG on LPL expression (representative bands in A and quantitative analysis in B) in mice with acute hyperlipemia

The results of western blot analysis showed that the expression of hepatic LPL in the hyperlipidemia group significantly decreased compared with the control group (vs control group, *P*<0.01, Figures 9A and 9B), leading to the reduction of TG clearance rate and the accumulation of liver lipids, thereby resulting in hyperlipidemia. Compared with the hyperlipidemia group, the expression of LPL significantly increased after administration of different doses of UAG (*P*<0.01, Figures 9A and 9B). The expression level of LPL in the high-dose UAG group as well as in the simvastatine group showed no significant difference from that in the control group (Figures 9A and 9B). The results indicated that UAG may up-regulate LPL expression in the liver, thereby promoting the elimination of TG to exert its lipid-lowering effect in the acute hyperlipidemia induced by poloxamer 407. 

## Discussion

Since the discovery of ghrelin as a gut peptide, the biological activity of its acylated form has attracted great attention. However, its unacylated form, UAG, was once considered to be inactive and has not received the attention it deserves. However, accumulating evidence strongly suggests that UAG has a number of functions in physiological and pathological conditions independently of AG (21-24). In these conditions, UAG supports or opposes the effects of AG. In the present study, we explored the effect of UAG on acute liver injury and hyperlipidemia and found that UAG exhibited certain hepatoprotective and lipid-lowering activities. 

As a toxic substance, CCl_4_ is a widely employed solvent to induce liver injury in animal models. CCl_4_ is metabolized by the cytochrome P450 enzyme to form reactive free radicals, resulting in hepatocyte damage and oxidative stress. The permeability of the damaged cell membrane increased a large amount of ALT, and AST poured into the blood, resulting in the increase of serum aminotransferase concentrations (25). In the present study, intraperitoneal injection of CCl_4_ significantly increased serum ALT and AST concentrations, indicating damage in hepatocytes. Simultaneously, the liver structure was destroyed, characterized by hepatomegaly and disordered hepatic lobule structure with cellular degeneration, necrosis, and inflammatory infiltration. Intraperitoneal injection of UAG significantly decreased serum ALT and AST concentrations and restored structure injury in the liver, showing a possible hepatoprotective effect in a dose-dependent manner. 

CCl_4_-triggered oxidative stress is another mechanism resulting in acute liver injury, manifested as the imbalance of preoxidants and anti-oxidants. SOD, a member of the free radical scavenging enzyme system, is recognized as a marker reflecting hepatic anti-oxidant capacity (26). As a product of lipid peroxidation, MDA indirectly reflects the degree of free radical damage to the liver (27). Excessive MDA will aggravate the damage of liver cells, leading to liver cell necrosis. Therefore, inflammatory factors such as TNF-α and IL-6 are released into the blood. TNF-α induces severe inflammatory response and hepatocyte apoptosis, therefore contributing to a variety of liver diseases (28). IL-6 is involved in the immune response of the body, promoting the occurrence of inflammation, and further promoting the aggravation of oxidative stress response (29). In our present study, there observed an elevation of serum IL-6, TNF-α, and hepatic MDA, as well as reduction of hepatic SOD concentration in the acute liver injury mice. After intraperitoneal injection of UAG, serum IL-6 and TNF-α and hepatic MDA decreased, while hepatic SOD concentration increased significantly compared with the liver injury group. The results demonstrated that UAG might inhibit oxidative stress and inflammatory reactions induced by CCl_4_, which contributed to its hepatoprotective effect.

NF-κB is an important transcription factor that participates in regulating inflammatory responses in a number of cell types (30-32). NF-κB is enormously generated in response to reactive oxygen species exposure, stress, and CCl_4_, which promotes the secretion of inflammatory cytokines and aggravates liver injury (33, 34). Our present study revealed that UAG effectively down-regulated NF-κB protein expression in the liver induced by CCl_4_. It was inferred that UAG might inhibit the activation of NF-κB, thereby suppressing the generation of IL-6 and TNF-α and inhibiting inflammatory reactions induced by CCl_4_. Taken together, UAG has a protective effect on acute liver injury mice, and the protective mechanism may be related to reducing liver oxidative stress and inhibiting inflammatory response.

Hyperlipidemia has become a worldwide public problem, because it may be a high risk factor for many chronic diseases, such as obesity, diabetes, metabolically associated fatty liver disease, and cardiovascular diseases (35). Poloxamer 407 is a commonly used nonionic surfactant that could cause hyperlipidemia and atherosclerosis in rodents (36). After poloxamer 407 intraperitoneally injected into mice, an increase of serum TC and TG and hepatic TG was observed in our present study. Moreover, hepatocyte steatosis was induced by poloxamer 407, manifested by fat vacuole deposits in the hepatocytes and disordered live lobes. However, intraperitoneal injection of UAG significantly decreased serum TC and TG and hepatic TG, showing a certain lipid-lowering activity induced by poloxamer 407.

The liver is the main organ that participates in fatty acid metabolism which is more susceptible to hyperlipidemia (37). Poloxamer-407 is widely distributed in the liver cells and inhibits LPL which is involved in lipid metabolism and transport, resulting in the generation of superoxide radicals (38). LPL is the key regulator of fatty acid uptake from triglyceride-rich lipoproteins. Studies (39) have shown that LPL not only regulates serum TG concentration but also plays an important role in fatty acid deposition in adipose tissue. Mice with reduced LPL concentration had higher fat mass and more insulin resistance (40), while mice with increased adipose LPL (41) had the opposite. In the present study, an elevation of serum ALT and AST was also observed in the hyperlipidemia group, indicating that poloxamer 407 induced hyperlipidemia as well as liver injury. Moreover, in the liver of the hyperlipidemia mice, MDA concentration increased, SOD concentration decreased, and LPL protein expression decreased, indicating that poloxamer 407 inhibited LPL in the liver, consequently triggering lipid peroxidation damage. Fortunately, UAG up-regulated the expression of LPL in the liver, as well as increasing hepatic SOD concentration and decreasing hepatic MDA concentration. The hepatoprotective and anti-oxidant effect of UAG might contribute to its lipid-lowering activity. 

Our present study primarily proposed the intervention effect of UAG on acute liver injury and hyperlipidemia for the first time. Exogenous UAG might reduce liver oxidative stress and inhibit inflammatory response, therefore contributing to its hepatoprotective and lipid-lowering activities. The present finding expanded the pharmacological spectrum of UAG. It is speculated that UAG might be potentially applied in lipid metabolism disorder and liver injury-related diseases such as obesity and metabolically associated fatty liver disease. However, the present study is a preliminary exploration of the pharmacological activity of UAG. A lot of work remains to be conducted to reveal the pharmacological and clinical application of UAG and its underlying mechanism.

Versus the control group, *P<0.05, **P<0.01; Versus the liver injury group, ^#^*P*<0.05, ^##^*P*<0.01; Versus UAG low dose group, ^&^*P*<0.01; Versus UAG medium dose group, ^△^*P*<0.05, ^△△^*P*<0.01.

UAG: Unacylated ghrelin

UAG: Unacylated ghrelin

Versus the control group, ^*^P<0.01; Versus the liver injury group, ^#^*P*<0.01; Versus UAG low dose group, ^&^*P*<0.05, ^&&^*P*<0.01; Versus UAG medium dose group, ^△^*P*<0.01.

UAG: Unacylated ghrelin; IL-6: Interleukin-6; TNF-α: Tumor necrosis factor-α

Versus control group, ^*^P<0.05, ^**^P<0.01; Versus liver injury group, ^#^*P*<0.05, ^##^*P*<0.01; Versus UAG low dose group, ^&^*P*<0.01; Versus UAG medium dose group, ^△^*P*<0.01.

UAG: Unacylated ghrelin; MDA: Malondialdehyde; SOD: Superoxide dismutase

Versus the control group, ^*^P<0.01; Versus the liver injury group,^ #^*P*<0.01; Versus UAG low dose group, ^&^*P*<0.05, ^&&^*P*<0.01; Versus UAG medium dose group, ^△^*P*<0.01.

UAG: Unacylated ghrelin; NF-κB: NF-kappa B

Versus control group, *P<0.05, **P<0.01; Versus hyperlipidemia group, ^#^*P*<0.05, ^##^*P*<0.01; Versus UAG low dose group, ^&^*P*<0.05, ^&&^*P*<0.01; Versus UAG medium dose group, ^△^*P*<0.05, ^△△^*P*<0.01.

UAG: Unacylated ghrelin

Bars:100 µm

UAG: Unacylated ghrelin

Versus control group, ^*^P<0.05, ^**^P<0.01; Versus liver injury group, ^#^*P*<0.05, ^##^*P*<0.01; Versus UAG low dose group, ^&^*P*<0.05, ^&&^*P*<0.01; Versus UAG medium dose group, ^△^*P*<0.05, ^△△^*P*<0.01

UAG: Unacylated ghrelin

Versus the control group,^*^P<0.05, ^**^P<0.01; Versus the liver injury group, ^#^*P*<0.01; Versus UAG low dose group, ^&^*P*<0.01.

UAG: Anacylated ghrelin; LPL: Lipoprotein lipase

## Conclusion

Our study revealed that intraperitoneal injection of UAG exhibited hepatoprotective and lipid-lowering effects on acute liver injury and hyperlipidemia, which are attributed to its anti-inflammatory and anti-oxidative activities. These observations demonstrated that UAG and its analog might be a potential target for the prevention of hyperlipidemia and liver injury-related diseases. Further investigations remain to explore the potential prospect of UAG for clinical application.

## Authors’ Contributions

Y G, B Q, H Z, X L, Y W, and M W conceived the study; Y G analyzed the data and prepared the draft manuscript; M Y and Y G critically revised the paper; M Y and Y G supervised the research; Y G, B Q, H Z, X L, Y W, M W, MY and Y G approval the final. 

## Conflicts of Interest

None of the authors has personal or financial conflicts of interest.
